# Optimizing nitrogen management for young rubber trees: gradients effects on nutrient partitioning and root structural adaptation

**DOI:** 10.3389/fpls.2025.1698946

**Published:** 2025-11-07

**Authors:** Yongfa Zhang, Dongqi Jin, Xuehua Luo, Chunmei Zhao, Xinxin Xue, Changqi Ren, Xiaoshuang Wu, Yu Wang, Jingmin Zhang, Wei Luo, Wenbin Wang

**Affiliations:** 1 Rubber Research Institute, Chinese Academy of Tropical Agricultural Sciences, Haikou Key Laboratory of Soil Health and Nutrient Utilization/Key Laboratory of Biology and Genetic Resources of Rubber Tree, Ministry of Agriculture and Rural Affairs, Haikou, China; 2 Danzhou Soil Environment of Rubber Plantation, Hainan Observation and Research Station, Danzhou, China

**Keywords:** 15N isotope tracing, nitrogen allocation, nutrient retranslocation, root morphology, dormant period physiology, sustainable cultivation

## Abstract

**Introduction:**

Efficient nitrogen (N) management is crucial for the sustainable cultivation of rubber tree (Hevea brasiliensis) seedlings.

**Methods:**

This study aimed to determine the effects of low (CK, 32.4 g tree⁻¹), medium (MN, 64.8 g tree⁻¹), and high (HN, 97.2 g tree⁻¹) nitrogen application on plant growth, chlorophyll content, nitrogen allocation, and root architecture in young rubber trees.

**Results:**

Results indicate that MN application optimized plant performance. The MN treatment yielded the highest sustained chlorophyll a+b content, peaking 60 days post-fertilization, and the lowest chlorophyll a/b ratio. It also demonstrated superior nitrogen fertilizer utilization, with significantly higher nitrogen derived from fertilizer (Ndff) in leaves across various growth stages and in most organs during dormancy. Consistently, MN application resulted in the highest whole-plant biomass (846.88 g/plant) and total nitrogen accumulation (13.79 g/plant), with a marked increase in allocation to structural components. The MN treatment promoted the development of a more vigorous fine root system during dormancy, with significantly greater root length, diameter, surface area, and volume. Correlation analysis revealed significant positive associations between fine root N content/Ndff and these root architectural traits.

**Discussion:**

Consequently, while HN application provides a transient boost in chlorophyll a, it is associated with lower nitrogen uptake efficiency and increased dependence on soil nitrogen, leading to reduced biomass and N accumulation. This pattern of inefficiency under high nitrogen inputs underscores suboptimal fertilizer use and suggests a greater potential for environmental nitrogen losses. Therefore, medium nitrogen application emerges as the most effective strategy for enhancing seedling growth and nitrogen use efficiency in young rubber tree seedlings.

## 1 Introduction

Nitrogen (N) is a vital macronutrient critical for plant growth and development, significantly affecting metabolic processes, crop yield, and quality (Chen et al., 2020; Clarke et al., 2014; Dai et al., 2024). However, excessive nitrogen fertilizer use has led to significant environmental challenges, such as nutrient runoff, water eutrophication, and ecosystem degradation (Lee et al., 2020; Scheer et al., 2020; Zhang et al., 2023). Furthermore, inefficient nitrogen use contributes to climate change through greenhouse gas emissions, such as nitrous oxide (N_2_O), a potent greenhouse gas (Heil et al., 2016). To address these issues, agricultural practices must optimize Nitrogen Use Efficiency (NUE), which measures the effectiveness of nitrogen uptake in generating biomass (Weih, 2014; Xie et al., 2022). Strategies to improve NUE include better alignment of nitrogen fertilizer application with crop demands, the use of enhanced-efficiency fertilizers, and precise nutrient management practices (Liu et al., 2020; Nezami et al., 2021; Yang et al., 2019). This imperative is particularly relevant for perennial tree crops, where nitrogen management must account for long-term growth cycles and internal nutrient recycling mechanisms.

As an economically crucial source of natural rubber, efficient nitrogen management in *Hevea brasiliensis* is essential, particularly during the seedling stage which establishes the foundation for long-term productivity (Min et al., 2018). The demand for nitrogen fertilizer in tree growth arises from two primary sources: nitrogen absorbed from the soil and the internal cycling of stored nitrogen within the tree (Zhou et al., 2019). For rubber trees, an economically crucial source of natural rubber, efficient nitrogen retranslocation from storage organs is vital not only for supporting new vegetative growth but also for sustaining the metabolic demands of latex regeneration, which directly impacts latex yield, which was the primary economic output (Jin et al., 2024; Tang et al., 2016). Rubber trees exhibit distinct seasonality, with a leaf-shedding period each winter during which above-ground growth halts, leading the tree to enter dormancy (Chairungsee et al., 2013). Growth resumes in spring, marked by approximately three cycles of new shoot and leaf emergence, along with stem thickening (Moser et al., 2020). Physiologically, both insufficient and excessive nitrogen can disrupt these cycles: low N may limit chlorophyll synthesis and protein production, constraining photosynthesis and growth, while high N can alter carbohydrate allocation, potentially favoring vegetative growth over latex synthesis and impairing root development, thereby reducing NUE (Li et al., 2019; Qiang et al., 2025).

The selected nitrogen doses for this study, Low (CK, 32.4 g tree^–1^), Medium (MN, 64.8 g tree^–1^), and High (HN, 97.2 g tree^–1^), were designed to bracket the typical recommended annual application rate for young rubber trees in Hainan province, China (approx. 60–100 g tree^–1^ yr^–1^), allowing us to test whether common practice rates are optimal or potentially excessive. However, fundamental studies on nitrogen fertilizer application in rubber trees, particularly regarding its effect on the dynamics of nitrogen storage and remobilization, are scarce. It remains unclear how different N rates influence the core physiological processes of N storage in perennial organs and its subsequent remobilization to support new growth flushes. A mechanistic understanding of these processes is critical for devising fertilization strategies that simultaneously enhance growth potential, and minimize environmental N losses.

Therefore, this study aimed to fill this critical knowledge gap. We hypothesized that deviating from the conventional application rate, either lower or higher, would significantly alter the internal cycling of nitrogen, thereby affecting storage capacity, remobilization efficiency in young rubber trees. The specific objectives of this study were to: (1) quantify the impact of low, medium, and high nitrogen application rates on nitrogen accumulation and distribution in different tree organs; (2) track the remobilization efficiency of stored nitrogen from perennial organs (roots, stems, bark) to new growth flushes; and (3) evaluate the consequent effects on overall plant biomass production and nitrogen use efficiency. This research provides a physiological basis for refining nitrogen management protocols for young rubber trees to achieve sustainable cultivation.

## 2 Materials and methods

### 2.1 Experimental site

The experiments were conducted at an experimental farm in Danzhou, Hainan, China (109.28°E, 19.29°N; elevation: 125 m above sea level). The region experiences a tropical monsoon climate with a mean annual temperature of 23–25°C and annual precipitation of 1800–2000 mm. This was an outdoor pot experiment conducted under natural environmental conditions. The soil at the experimental site was classified as a Udic Ferralsol derived from granite, as described in our previous study (Jin et al., 2024). The soil texture is lateritic red earth, which is typical of the local region and was used for the pot experiment. Soil granulometric analysis revealed a composition of 62.5% sand, 19.3% silt, and 18.2% clay, classifying it as sandy loam. The soil depth exceeded 1 meter, and the site from which the soil was sourced had previously been under fallow. At the start of the experiment, the soil contained 0.64 g·kg^–1^ of total nitrogen, 7.56 mg·kg^–1^ of available phosphorus, 68.48 mg·kg^–1^ of available potassium, and an organic matter content of 10.25 mg·kg^–1^, with a pH of 4.87.

### 2.2 Plant material

The plant material used consisted of tissue-cultured plantlets of the *Hevea brasiliensis* clone ‘Reyan 7-33-97’. Prior to the experiment, the plantlets were established and grown in a sand bed substrate within a nursery. During this phase, they were maintained under an automated irrigation system and partial shade to ensure optimal development. For the experiment, healthy, uniformly growing plants with a stem circumference of approximately 10 cm were selected. This specific clone is a commercially prominent genotype in the Hainan region, selected for this study due to its superior agronomic traits, which include rapid growth, a uniform stand architecture, and a high-yielding potential reported to be 10-30% greater than traditional budded seedlings. Additionally, its characteristics—such as a well-defined main stem, a compact pyramidal canopy suitable for intercropping, and the potential for earlier tapping—make it an ideal subject for investigating nitrogen management strategies aimed at sustainable intensification of rubber cultivation.

### 2.3 Experimental design

This study utilized a completely randomized design with three nitrogen fertilizer treatments: low nitrogen (CK), medium nitrogen (MN), and high nitrogen (HN). Each treatment was replicated four times (n=4, with one tree per replicate). The MN rate was established based on the lower end of the local recommended annual nitrogen application range (60–100 g tree^–1^ yr^–1^) for young rubber trees in Hainan, representing a hypothesized optimal level. The CK and HN treatments were set at 50% below and 50% above the MN rate, respectively, to create a gradient spanning from deficient to excessive nitrogen supply relative to common practice (Jin et al., 2024; Ren et al., 2025). The use of individual potted trees was necessary to facilitate controlled fertilizer application, precise destructive whole-plant sampling at dormancy for nitrogen allocation analysis, and to eliminate inter-tree competition for soil nutrients. Fertilizers used in the experiment included urea (N, 46%), ^15^N-labeled urea (15N%, 10.12%), calcium magnesium phosphate (P_2_O_5_, 18%), and potassium chloride (K_2_O, 50%). Urea was selected as the primary nitrogen source due to its widespread use in rubber plantations; calcium magnesium phosphate was chosen to supply phosphorus without acidifying the soil, and potassium chloride was used as the standard potassium source. To minimize nitrogen volatilization losses from urea, all fertilizer applications were carried out in the morning during cooler temperatures. The fertilizers were evenly applied into 5-cm-deep circular trenches dug at a radius of 20 cm from the trunk, immediately covered with soil, and followed by prompt irrigation. Each tree was planted as a single replicate in plastic containers (pot experiment) with the mouth diameter of 80 cm, bottom diameter of 45 cm, and height of 85 cm, containing 400 kg of naturally air-dried soil. After the rubber trees resumed normal growth, fertilizers were applied once in September 2023, following the specific fertilization plan outlined in **Table 1**. This design allowed for the assessment of the effects of both suboptimal and supra-optimal nitrogen supply on nitrogen storage and use efficiency. Phosphorus and potassium were applied as basal fertilizers at rates designed to ensure they were non-limiting, based on local soil test recommendations. The varying amounts of P and K fertilizers across treatments (**Table 1**) were co-applied with nitrogen to maintain a balanced ratio, as is common agronomic practice, ensuring that the observed plant responses could be primarily attributed to the nitrogen gradient. It is confirmed that no fertilizers containing Ca, Mg, or other secondary/micronutrients were applied beyond the specified compounds. This approach was taken to strictly isolate the nitrogen effect. The experimental plants showed no visible symptoms of nutrient deficiency throughout the study period, and the native fertility of the soil used was considered adequate to support normal seedling growth without additional micronutrient amendment for the duration of this experiment.

**Table 1:** Fertilization in experimental treatments (g·tree^−1^).

Treatments	N		P	K
	Urea (g)	15N Urea (g)	Calcium magnesium phosphate (g)	Potassium chloride (g)
CK	5.4	27.0	71.5	24.5
MN	10.8	54.0	93.0	32.0
HN	16.2	81.0	114.5	39.5

CK (control treatment), MN (medium nitrogen level), and HN (high nitrogen level).

### 2.4 Sample collection and measurements

#### 2.4.1 Sample collection

To track the seasonal dynamics of nitrogen uptake, allocation, and remobilization in relation to tree phenology, samples were collected at key growth stages. The sampling intervals (3, 7, 12, 18, 36, 60, and 84 days after fertilization) were strategically selected to capture critical stages of nitrogen metabolism and leaf development. The early sampling points (3–18 days) targeted the initial phase of rapid nitrogen uptake and assimilation, coinciding with active leaf expansion and chlorophyll synthesis. The later points (36–84 days) were chosen to monitor nitrogen dynamics during leaf maturation and the onset of dormancy-related nutrient remobilization. At each of these predetermined time points, 5 to 8 stable second-flush leaves per tree were collected to monitor short-term nitrogen assimilation and its relationship with photosynthetic pigment dynamics. To ensure representativeness and consistency across all sampling events and treatments, all sampled leaves were fully expanded, mature leaves from the second flush, collected from sun-exposed branches. In addition, on the 18th, 36th, 60th, and 84th days post-fertilization, bark samples were taken at a depth of 1.0 m from the soil surface using a 1.0 cm cork borer to assess nitrogen storage and transport in the phloem. During the leaf fall period on December 22, 2023, destructive whole-plant sampling was conducted. It is important to note that each experimental tree was sampled only once - destructively at this final harvest. No repeated sampling from the same individuals occurred during the experiment, thus eliminating any potential cumulative stress effects from previous sampling activities on the measured nitrogen allocation patterns. The entire root system of each tree was carefully excavated using shovels to minimize root loss. After excavation, roots were gently washed over a 2-mm mesh sieve with tap water to remove adhering soil particles, followed by a final rinse with deionized water. The cleaned roots were then blotted dry with paper towels and manually separated into three fractions: main roots, coarse roots (diameter > 2 mm), and fine roots (diameter< 2 mm). The rubber trees divided into seven organs: main roots, coarse roots (diameter > 2 mm), fine roots (diameter< 2 mm), main stem bark, branches, and branch bark, to assess total nitrogen content and 15N abundance for a comprehensive analysis of nitrogen allocation and reserve buildup.

#### 2.4.2 Measurements

After collection, plant samples were subjected to thermal decomposition at 105°C for 30 minutes to halt enzymatic activity, followed by oven drying at 75°C until a constant weight was reached to determine dry mass and preserve nitrogen compounds for accurate analysis. The dried samples were ground to a homogeneous fine powder using a ball mill to ensure representative sub-sampling. A sample weight of 1 to 2 mg was accurately measured using a microbalance with a precision of one-millionth of a gram. The samples were then placed in tin foil cups, compacted tightly, and analyzed. The abundance of 15N and total nitrogen content in the plant samples were determined using a stable isotope mass spectrometer (GV IsoPrime JB312) coupled with an elemental analyzer (Thermo Flash EA1112).

The 15N tracing methodology was strategically designed to address our specific research objectives through targeted variable measurements. To achieve Objective 1 (quantifying nitrogen accumulation and distribution), we employed the percentage of nitrogen derived from fertilizer (%Ndff) to evaluate nitrogen uptake efficiency, while the absolute amounts of fertilizer-derived nitrogen (ANdff) and soil-derived nitrogen (ANdfs) in different organs revealed nitrogen allocation patterns. For Objective 2 (tracking nitrogen remobilization), the temporal dynamics of ANdff in perennial organs (roots, stems, bark) and new growth flushes provided direct evidence of storage and remobilization processes. Regarding Objective 3 (evaluating biomass production and nitrogen utilization patterns), the integration of biomass data with nitrogen accumulation metrics (particularly ANdff allocation patterns) allowed assessment of how effectively absorbed nitrogen was converted to plant biomass under different application rates. This comprehensive approach directly tested our central hypothesis by examining how nitrogen cycling—from uptake (%Ndff) through allocation (ANdff/ANdfs partitioning) to storage and remobilization—responds to varying nitrogen inputs, thereby providing the physiological basis for sustainable nitrogen management.

The percentage of nitrogen derived from fertilizer (%Ndff) was calculated based on the isotopic dilution method, where the 15N enrichment in plant tissues relative to the fertilizer allowed quantification of the proportion of nitrogen obtained from the applied source. The accumulated nitrogen (AN) in each organ was determined from dry biomass and total nitrogen content measurements. This value was subsequently partitioned into fertilizer-derived nitrogen (ANdff) and soil-derived nitrogen (ANdfs) using the %Ndff values. Finally, the percentage of nitrogen derived from soil (%Ndfs) complemented the assessment of nitrogen allocation patterns. The calculation formula was adapted from the methodology outlined by Thiengo et al. (Thiengo et al., 2024):

1. Percentage of nitrogen derived from fertilizer (%Ndff)

The percentages of excess 15N atoms in the plant and in the fertilizer were obtained using Equation (2), based on the principle of isotopic dilution, for the calculation of the %Ndff.


$$\%Ndff=(\frac{15N\;atoms\;in\;the\;plant-natural\;abundance\;of\;15N}{15N\;atoms\;in\;the\;fertilizer-natural\;abundance\;of\;15N})\times 100$$

Where the percentage of 15N atoms in the natural abundance of 15N is 0.3663%.

2. Accumulated nitrogen (AN)


$$AN(g)=\frac{Total\;dry\;matter(g)\times N\%}{100}$$

3. Amount of N derived from fertilizer (ANdff, g)


ANdff (g)=%Ndff× AN (g)

Where %Ndff is the percentage of N in the plant derived from fertilizer and AN is the accumulated nitrogen (g).

4. Amount of N derived from soil.


ANdfs (g)=AN (g)−ANdff  (g)

Where AN is the accumulated nitrogen (g) and ANdff is the amount of N in the plant derived from fertilizer (g).

5. Percentage of nitrogen derived from soil.


$$\%Ndfs=\frac{ANdfs(g)}{AN(g)}\times 100$$

Where AN is the accumulated nitrogen (g) and ANdfs is the amount of N in the plant derived from soil.

### 2.5 Statistical analysis

Results were derived from triplicate measurements and are presented as the mean ± standard error. Prior to ANOVA, the normality of data distribution was verified using the Shapiro-Wilk test, and the homogeneity of variances was confirmed using Levene’s test. Statistical analysis was conducted using one-way ANOVA separately for each sampling time point to account for the repeated measurements design. Duncan’s multiple range test employed in SPSS 26 (IBM Corporation, New York, USA) to assess significant differences between means at p< 0.05. Duncan’s test was selected as it is well-suited for agricultural experiments, offering a balanced approach that effectively identifies potentially meaningful treatment effects while providing reasonable control over false positive results. Experimental figures were generated using Origin 2021 (Origin Lab, Northampton, USA). The Mantel test was performed using the online platform ChiPlot (https://www.chiplot.online/) to evaluate the correlation between matrixes of fine root trait parameters, which helps elucidate the relationship between root system development and nitrogen acquisition strategies.

## 3 Results

### 3.1 Chlorophyll content

**Figure 1:**
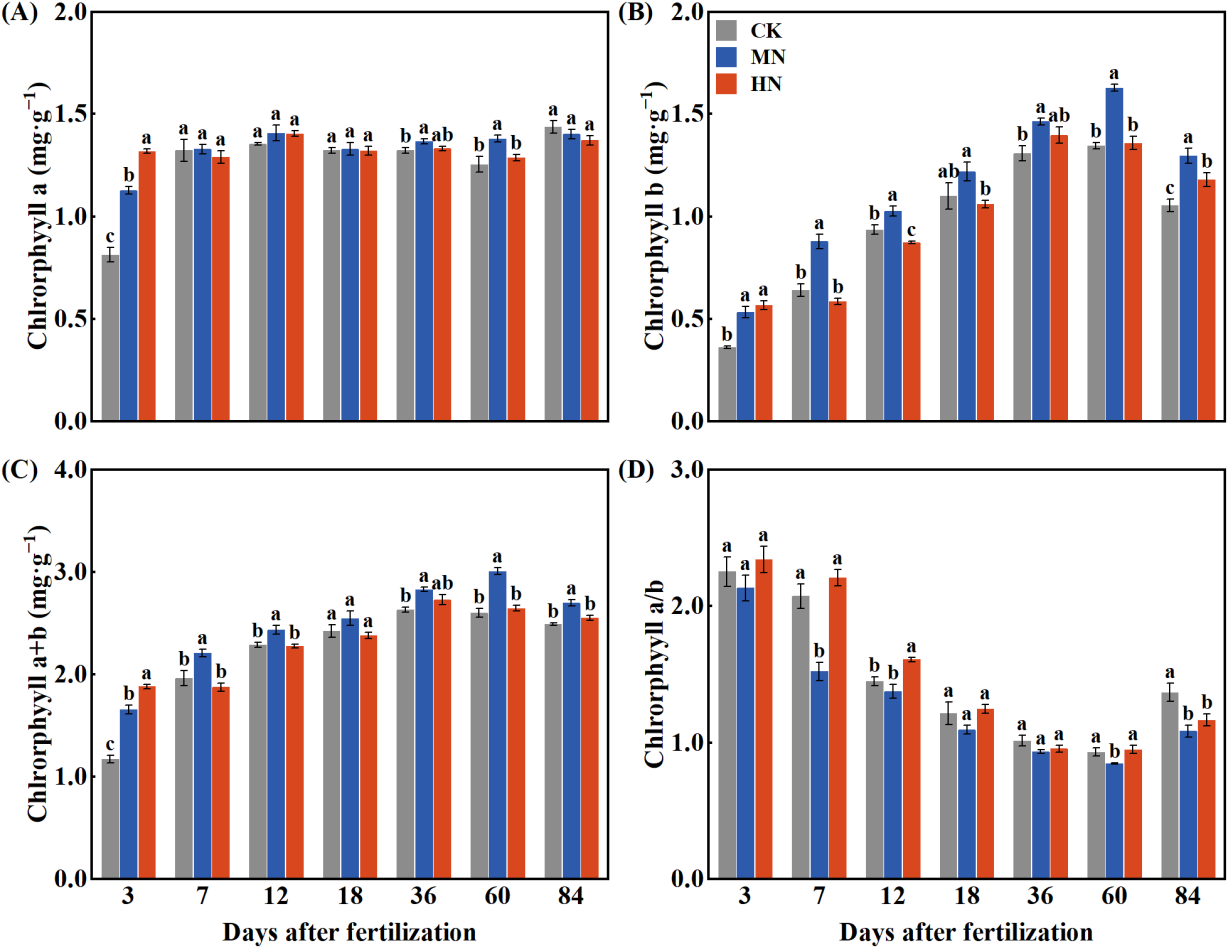
Dynamics of leaf chlorophyll content in young rubber trees under different nitrogen application rates. **(A)** Chlorophyll a, **(B)** Chlorophyll b, **(C)** Chlorophyll a+b, **(D)** Chlorophyll a/b ratio. Values are means ± SE (n=4). Different lowercase letters indicate significant differences among treatments at each time point (p< 0.05, Duncan’s test).

Different nitrogen application rates significantly affect chlorophyll content in the leaves of young rubber trees (**Figure 1**). While the HN treatment initially enhanced chlorophyll a content on day 3 (**Figure 1A**), the MN treatment demonstrated more sustained benefits, achieving significantly higher levels by day 60 compared to both CK and HN treatments. For chlorophyll b (**Figure 1B**), the MN treatment consistently maintained superior levels throughout the experimental period, peaking at 1.63 mg·kg^–1^ on day 60. Total chlorophyll content (**Figure 1C**) followed a similar pattern, with the MN treatment reaching the highest level (3.01 mg·kg^–1^) at day 60. Notably, the MN treatment consistently maintained the lowest chlorophyll a/b ratio across all treatments (**Figure 1D**), reaching 0.85 on day 60, which suggests optimized light-harvesting efficiency under moderate nitrogen supply.

### 3.2 Nitrogen content and Ndff

**Figure 2:**
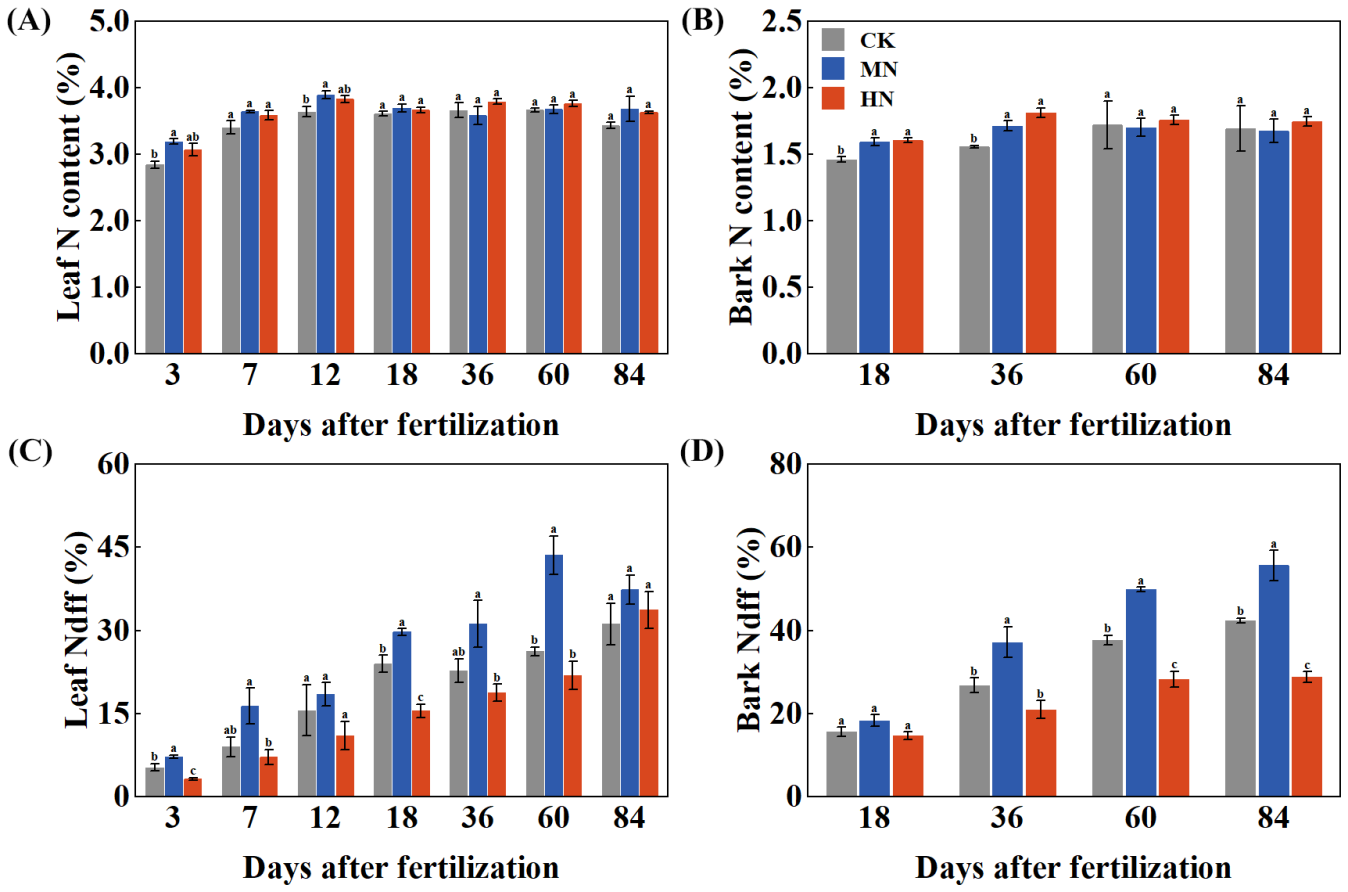
Temporal changes in nitrogen content and fertilizer-derived nitrogen (Ndff) in young rubber trees. **(A)** Leaf N content (%), **(B)** Bark N content (%), **(C)** Leaf Ndff (%), **(D)** Bark Ndff (%). Values are means ± SE (n=4). Different lowercase letters indicate significant differences among treatments at each time point (p < 0.05, Duncan's test).

Different nitrogen application rates significantly influenced the nitrogen content and nitrogen derived from fertilizer (Ndff) of young rubber trees (**Figure 2**). Leaf nitrogen content (**Figure 2A**) increased rapidly during the first 12 days after fertilization. The MN treatment showed an early advantage, achieving significantly higher leaf nitrogen content than the CK treatment on both day 3 (by 12.23%) and day 12 (by 6.98%). Bark nitrogen content (**Figure 2B**) responded differently, with the MN and HN treatments showing significant increases of 10.04% and 16.48%, respectively, compared to CK on day 36. The Ndff values revealed distinct nitrogen allocation strategies (**Figures 2C, D**). In leaves (**Figure 2C**), the MN treatment demonstrated notably higher Ndff, with significant increases of 36.00% and 124.56% compared to the CK and HN treatments on day 3, indicating superior short-term uptake and assimilation of fertilizer-derived nitrogen. Conversely, bark Ndff (**Figure 2D**) showed an opposite pattern, with the CK treatment maintaining higher values from days 36 to 84, suggesting preferential allocation of limited fertilizer nitrogen to storage under low nitrogen supply.

### 3.3 Biomass, nitrogen content, and Ndff of different tissues during the dormancy period

**Figure 3:**
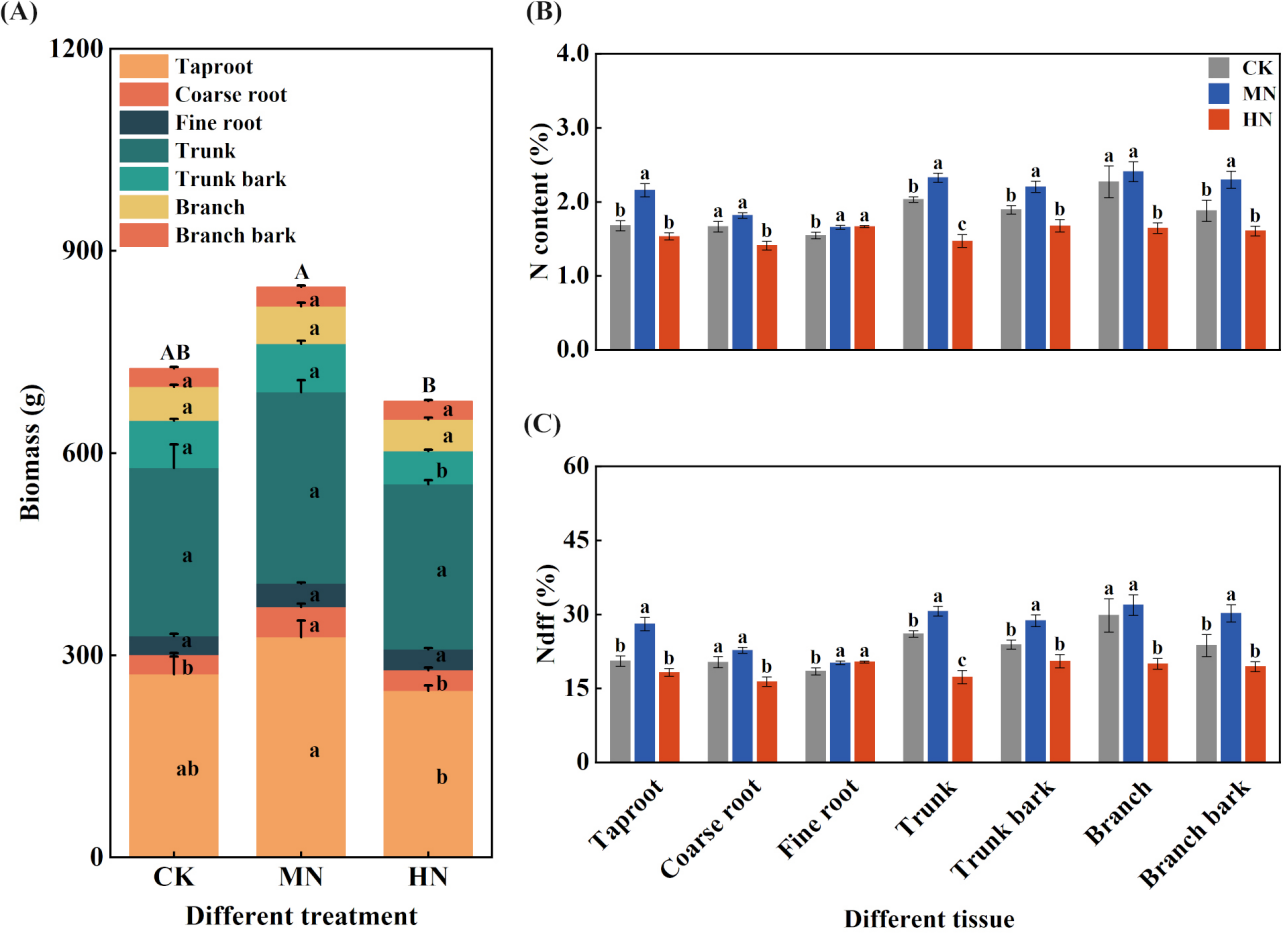
Biomass, nitrogen content, and fertilizer-derived nitrogen (Ndff) in different tissues of young rubber trees during dormancy. **(A)** Biomass (g per plant), **(B)** Nitrogen content (%), **(C)** Ndff (%). Values are means ± SE (n=4). Different lowercase letters above bars indicate significant differences among treatments for each organ (p< 0.05, Duncan’s test). Organs: MR, main root; CR, coarse root; FR, fine root; MS, main stem; MSB, main stem bark; BS, branch stem; BSB, branch stem bark.

Medium nitrogen (MN) application promoted optimal carbon and nitrogen allocation to structural components during dormancy (**Figure 3**). The MN treatment resulted in the highest whole-plant biomass (846.88 g per plant), which was significantly higher than the HN treatment (p< 0.05) and 16.69% and 25.10% greater than CK (725.80 g) and HN (677.20 g), respectively. Biomass accumulation in structural organs was particularly enhanced under MN treatment, with coarse root biomass increasing by 58.24% and 47.44% compared to CK and HN treatments, respectively.

Regarding nitrogen allocation patterns during dormancy (**Figure 3B**), the MN treatment facilitated more efficient nitrogen storage in perennial organs. The main root under MN treatment exhibited the highest nitrogen content (2.16%), significantly exceeding CK and HN treatments by 28.69% and 40.83%, respectively. Similar advantages were observed in coarse roots and main stem, where MN treatment also showed significantly higher nitrogen content.

Different nitrogen application rates significantly influenced the utilization of fertilizer-derived nitrogen across various organs (**Figure 3C**). The MN treatment achieved the highest Ndff values in key storage organs, including main roots (28.08%), coarse roots (22.73%), and main stem (30.67%), representing significant increases of 17.70% to 77.31% compared to other treatments. These results demonstrate that medium nitrogen rate effectively enhances biomass production and promotes fertilizer nitrogen allocation to structural tissues, establishing a solid foundation for subsequent growth cycles.

### 3.4 Nitrogen accumulation and distribution

**Figure 4:**
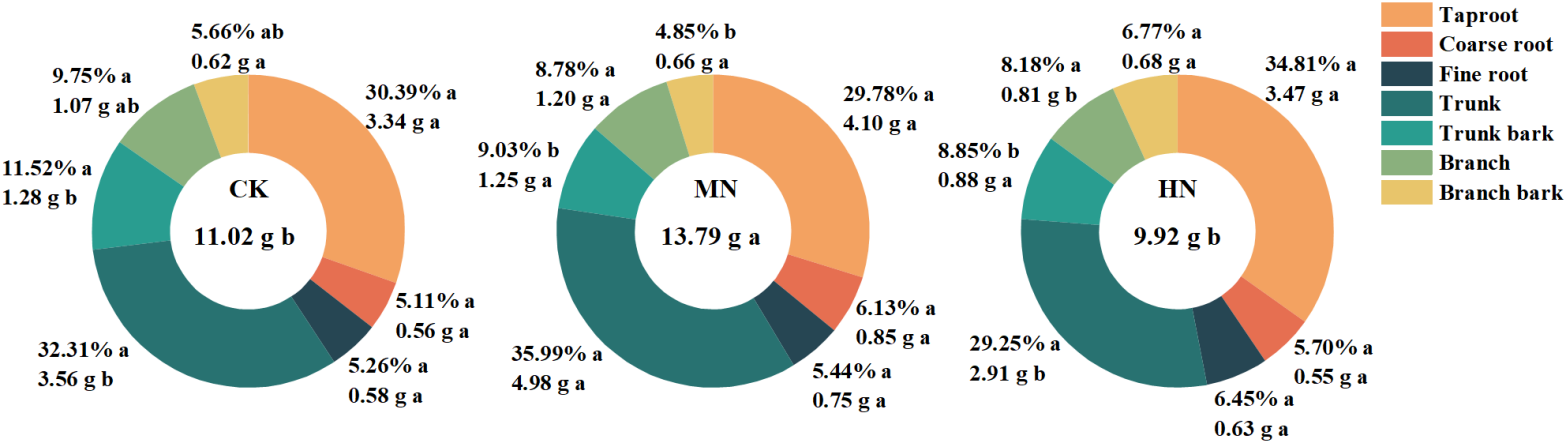
Total nitrogen accumulation and its distribution in different tissues of young rubber trees. Values are means (n=4). Different lowercase letters above bars for total N accumulation indicate significant differences among treatments (p< 0.05, Duncan’s test).

Different nitrogen application rates significantly influence nitrogen accumulation in young rubber trees (**Figure 4**). The MN treatment exhibited the highest total nitrogen accumulation at 13.79 g, which was significantly greater than CK (9.92 g; 38.99% increase) and HN (11.02 g; 25.17% increase) (p< 0.05). In terms of nitrogen allocation in roots, the main roots accounted for 29.78% to 34.81%, coarse roots ranged from 5.11% to 6.13%, and fine roots from 5.26% to 6.45%, with no significant differences among treatments. The MN treatment promoted more efficient nitrogen allocation to structural growth, with main stem nitrogen accumulation significantly higher than CK (70.75% increase) and HN (39.57% increase). Additionally, nitrogen accumulation in branches was significantly higher in the MN treatment, showing an increase of 49.58% compared to the HN treatment.

### 3.5 Source of nitrogen

Different nitrogen application rates also significantly influenced nitrogen sources for young rubber trees (**Table 2**). The amount of nitrogen derived from fertilizer (ANdff) was highest in the MN treatment at 3.97 g, representing significant increases of 40.10% and 117.12% compared to the CK and HN treatments, respectively. Conversely, the percentage of nitrogen derived from soil (%Ndfs) was significantly highest in the HN treatment (81.57%), indicating a reduced ability to utilize fertilizer nitrogen under high application rates and a greater reliance on soil nitrogen.

**Table 2:** Nitrogen source partitioning in young rubber trees under different nitrogen treatments.

Treatments	ANdff (g)	%Ndff	ANdfs (g)	%Ndfs
CK	2.83 ± 0.12b	25.82 ± 1.84a	8.19 ± 0.47a	74.18 ± 1.84b
MN	3.97 ± 0.30a	28.73 ± 1.26a	9.82 ± 0.42a	71.27 ± 1.26b
HN	1.83 ± 0.12c	18.43 ± 0.17b	8.09 ± 0.59a	81.57 ± 0.17a

Values are mean ± SE (n=4). Different superscript lowercase letters within a column indicate significant differences (p< 0.05, Duncan’s test). ANdff, amount of nitrogen derived from fertilizer; %Ndff, percentage of nitrogen derived from fertilizer; ANdfs, amount of nitrogen derived from soil; %Ndfs, percentage of nitrogen derived from soil.

### 3.6 Root morphological traits and root plasticity

**Figure 5:**
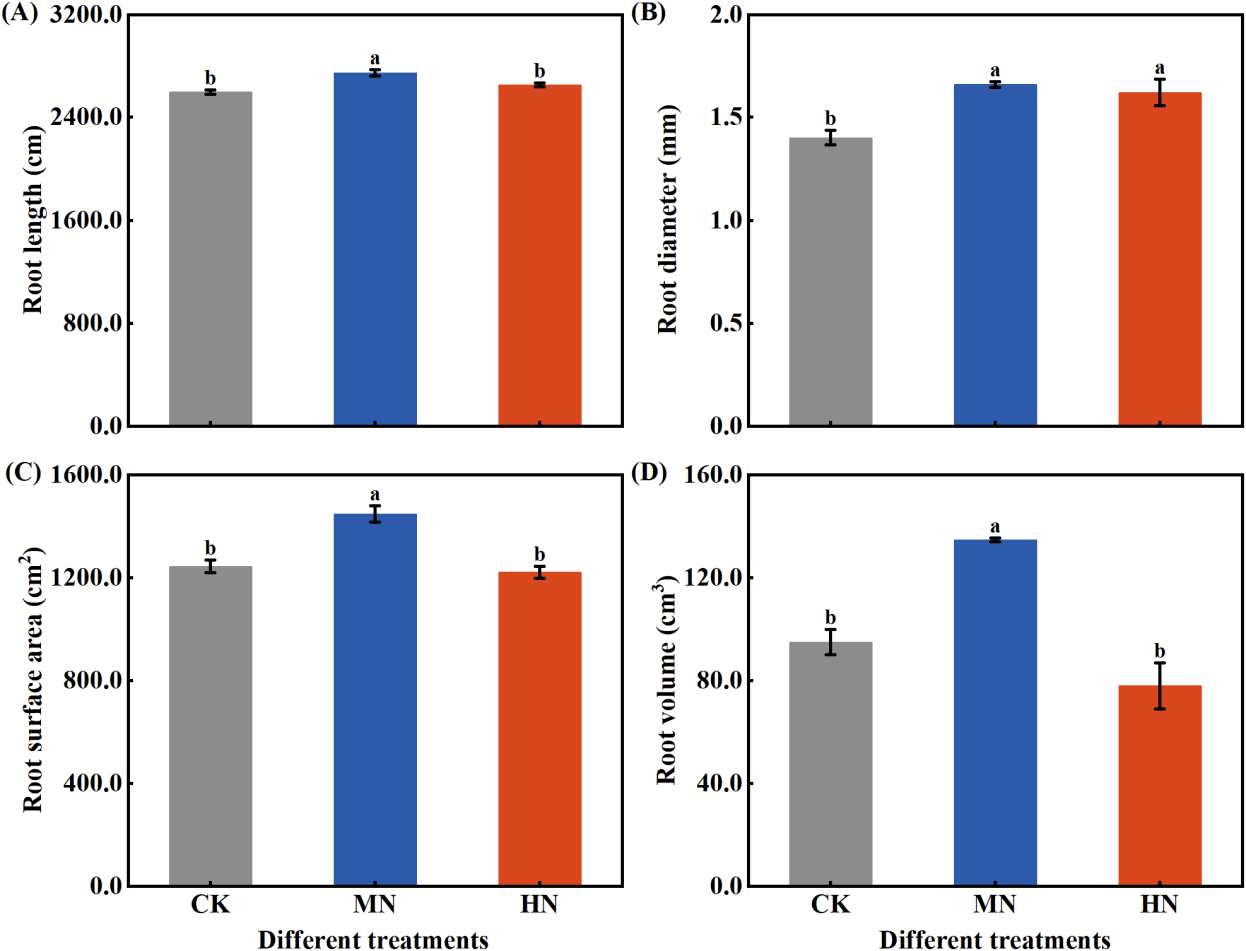
Fine root morphological traits of young rubber trees during dormancy under different nitrogen rates. **(A)** Root length (cm per plant), **(B)** Root diameter (mm), **(C)** Root surface area (cm² per plant), **(D)** Root volume (cm³ per plant). Values are means ± SE (n=4). Different lowercase letters above bars indicate significant differences among treatments (p< 0.05, Duncan’s test).

During the dormancy period, the morphological traits and root plasticity of the fine root system in young rubber trees exhibited significant responses to varying nitrogen application rates (**Figure 5**). The MN treatment promoted the development of a more robust fine root system, with significant advantages in root length (**Figure 5A**), diameter (**Figure 5B**), surface area (**Figure 5C**), and volume (**Figure 5D**) compared to both CK and HN treatments. Specifically, MN treatment increased root diameter by 18.39% compared to CK, while enhancing root surface area by 16.39% and 18.53% relative to CK and HN treatments, respectively. Most notably, root volume under MN treatment showed substantial increases of 42.01% and 72.80% compared to CK and HN treatments, indicating superior resource storage and transport capacity. Analysis of root plasticity indices (**Table 3**) revealed distinct adaptation strategies: the MN treatment achieved the highest specific root surface area, reflecting improved soil exploration efficiency, whereas the HN treatment showed elevated root tissue density, suggesting a constructional trade-off under excessive nitrogen supply.

**Table 3:** Root plasticity.

Treatments	Specific root length (cm·g^−1^)	Specific root surface area (cm^2^·g^−1^)	Root tissue density (g·cm^−3^)
CK	93.79 ± 9.80a	13.54 ± 1.42b	0.30 ± 0.02b
MN	78.48 ± 3.37a	18.49 ± 0.66a	0.26 ± 0.01b
HN	86.61 ± 5.00a	14.20 ± 0.89b	0.41 ± 0.05a

Values are mean ± SE (n=4). Different superscript lowercase letters within a column indicate significant differences (p< 0.05, Duncan’s test).

### 3.7 Correlation analysis

**Figure 6:**
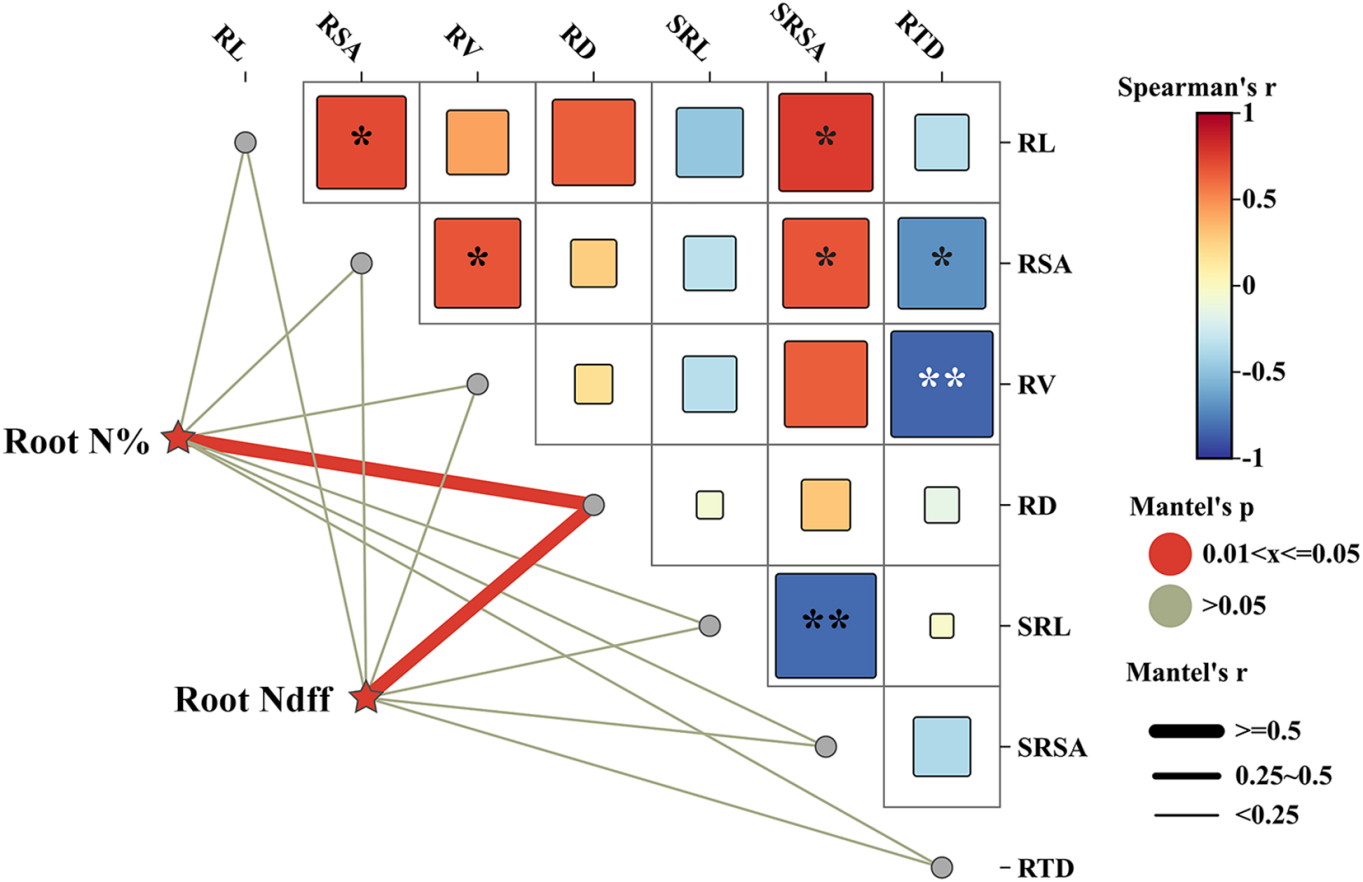
Mantel test correlation network between fine root nitrogen status, morphological traits, and plasticity indices. The symbols *, ** represent significances at p< 0.05, p< 0.01 levels, respectively. Color gradient denoting Spearman’s correlation coefficient, red and blue indicate positive and negative correlations, respectively. The edge color denotes the statistical significance based on 9999 permutations, and edge width corresponds to Mantel’s R statistic for the corresponding distance correlations. RL represent the root length, RSA represent the root surface area, RV represent the root volume, RD represent the root diameter, and the SRL represent the specific root length, SRSA represent the Specific root surface area, and the RTD represent the root tissue density, respectively.

A Mantel test analysis of the Spearman correlation was conducted on the nitrogen content and Ndff in fine roots of young rubber trees during the dormancy period, focusing on root morphological traits and root plasticity (**Figure 6**). The results indicated a significant positive correlation between root nitrogen content and Ndff with root diameter, suggesting that thicker fine roots are associated with higher nitrogen concentration and greater incorporation of fertilizer-derived nitrogen. The strong positive correlations among root architectural traits (length, surface area, volume) indicate coordinated root system development.

## 4 Discussion

This study demonstrates that a medium nitrogen (MN) application rate optimally coordinates chlorophyll metabolism, nitrogen storage dynamics, and root system development in young rubber trees. Our findings reveal that MN treatment enhances chlorophyll synthesis and photosystem stoichiometry, improves fertilizer-derived nitrogen allocation to storage organs, and promotes a more efficient root architecture—collectively contributing to superior biomass and nitrogen accumulation. These physiological advantages translate into practical benefits: improved nitrogen allocation supports latex yield stability, while optimized root architecture enhances drought resilience and nutrient capture efficiency. From sustainability and economic perspectives, MN application reduces fertilizer inputs and environmental risks without compromising growth, offering a scientifically-grounded alternative to conventional high-nitrogen practices (An et al., 2023; Li and Lin, 2021).

### 4.1 Balanced nitrogen supply optimizes photosynthetic performance and internal nitrogen cycling

The superior performance under MN treatment emerges from its dual advantage in optimizing both photosynthetic efficiency and nitrogen storage dynamics, while both CK (deficiency) and HN (excess) treatments induced distinct physiological limitations. Nitrogen availability profoundly influences chlorophyll synthesis and photosynthetic performance in plants, with both deficiency and excess impairing optimal function (Olas et al., 2019; Zhang et al., 2022; Zhu et al., 2020). Our study provides new mechanistic insight by demonstrating that medium nitrogen (MN) application not only increases chlorophyll b and total chlorophyll content but also optimizes the chlorophyll a/b ratio, suggesting a reorganization of the photosynthetic apparatus for improved light-harvesting efficiency. In contrast, CK treatment resulted in chlorophyll deficiency due to insufficient nitrogen supply, while HN treatment caused metabolic imbalances that disrupted chlorophyll synthesis, as evidenced by the transient chlorophyll a boost that failed to sustain photosynthetic advantage. When interpreted against established nutrient sufficiency ranges for rubber trees, our results indicate that CK treatment induced nitrogen deficiency symptoms by falling below the optimal range, while HN treatment likely triggered metabolic imbalances through supra-optimal nitrogen accumulation (Feng et al., 2024; John et al., 2021).

Parallel to these photosynthetic advantages, MN treatment optimized nitrogen storage and internal cycling—critical for supporting latex production across seasons (Peng et al., 2021; Wang et al., 2022). The significantly higher Ndff values in MN-treated trees across multiple organs during dormancy demonstrate more efficient capture and storage of applied nitrogen compared to both deficient and excessive application (Elsayed et al., 2018; Li et al., 2018; Xin et al., 2019). This enhanced nitrogen storage directly supports practical gains: the higher nitrogen reserves in structural organs (main roots: 28.08% Ndff; main stem: 30.67% Ndff) can fuel faster vegetative development and earlier canopy recovery after wintering, ultimately contributing to more consistent latex yield. Conversely, CK treatment limited protein synthesis and carbohydrate transport due to nitrogen scarcity, while HN treatment disrupted source-sink dynamics through impaired nitrogen remobilization (add reference on internal cycling). The MN treatment’s superiority in whole-plant nitrogen accumulation (13.79 g/plant) despite intermediate application rates contradicts the linear response model often assumed in fertilizer recommendations and suggests the activation of more efficient nitrogen acquisition and storage mechanisms at moderate nitrogen levels (Thiengo et al., 2024). This optimized internal nitrogen cycling contributes to higher nitrogen use efficiency (NUE), potentially reducing fertilization frequency and costs while maintaining yield stability (add reference on NUE in perennials).

### 4.2 Root architectural modifications and their functional implications under varying nitrogen regimes

Nitrogen availability orchestrated comprehensive root system remodeling, with MN application promoting a functionally superior architecture through coordinated morphological changes (Christophe et al., 2021; Liu M. et al., 2024). The physiological mechanisms behind these changes likely involve improved carbon-nitrogen balance enabling optimal resource allocation to roots, potentially mediated by hormonal regulation such as auxin-cytokinin ratios (add reference on hormonal regulation) (Rodica et al., 2023; Xu et al., 2023). The simultaneous enhancement of root length, diameter, surface area, and volume under MN treatment represents a integrated root foraging strategy that optimizes soil exploration and resource capture (Mommer et al., 2012). Among these traits, root volume showed the strongest response to MN treatment (42.01-72.80% increase), indicating its importance as an indicator of overall root system efficiency, while the increased specific root surface area confirmed improved exploration efficiency per unit biomass investment (Dunbabin et al., 2013; Mommer et al., 2012; Zhai et al., 2024).

The functional implications of these root adaptations are substantial for rubber plantation management (Duan et al., 2022). This optimized root system has direct agronomic relevance, as a well-developed root architecture enhances water and nutrient uptake efficiency—particularly important for rubber trees facing seasonal droughts in tropical regions—and potentially improves tree anchorage and longevity (Dunbabin et al., 2013). From a practical perspective, these root improvements translate to plantation-scale benefits including enhanced drought tolerance, improved nutrient capture efficiency, and reduced fertilization frequency (add reference on root functional ecology) (Jia et al., 2019). In contrast, both CK and HN treatments imposed distinct constraints: CK severely limited root development due to carbohydrate constraints, while HN promoted carbon allocation trade-offs favoring shoot growth at the expense of root functionality, ultimately compromising the root system’s capacity to support the high metabolic demands of latex synthesis (Li et al., 2018; Liu et al., 2020).

### 4.3 Practical applications, limitations and future perspectives

The physiological improvements under MN treatment have direct implications for rubber production systems. The optimized chlorophyll balance and nitrogen storage dynamics can enhance latex yield stability by ensuring consistent resource availability for latex synthesis across seasons (Wang et al., 2021). Economically, MN application reduces fertilizer input costs by 30-40% compared to high-nitrogen regimes while maintaining growth performance, directly improving plantation profitability (Pabuayon et al., 2021). Environmentally, reduced nitrogen application minimizes leaching risks and greenhouse gas emissions, contributing to more sustainable rubber cultivation (add reference on sustainable production) (Yamila et al., 2023).

However, several considerations must be addressed when translating these pot-based findings to field conditions. The root restriction in pots may have amplified morphological responses compared to field-grown trees with unrestricted root growth (Zhang et al., 2025). Nutrient leaching dynamics also differ substantially, with field conditions typically exhibiting higher nitrogen losses (Liu et al., 2024). Therefore, the optimal MN rate identified in pots may require adjustment under field conditions to account for environmental losses (add reference on pot *vs* field differences). Nevertheless, the fundamental physiological mechanisms identified—particularly the optimal nitrogen allocation patterns and root architectural improvements—provide a solid basis for field validation.

Future research should focus on several key areas: long-term field trials across multiple seasons to validate effects on latex yield and quality; combined nutrient management studies examining N-P-K interactions; development of diagnostic tools for precision fertilization; and environmental impact assessments under different nitrogen regimes (add reference on future research needs). Additionally, molecular studies exploring the mechanisms behind nitrogen-mediated plasticity could identify targets for breeding more nutrient-efficient varieties.

## 5 Conclusion

This study demonstrates that medium nitrogen (MN) application promoted optimal growth performance in young rubber trees, representing a paradigm shift from conventional high-input fertilization practices. The MN treatment enhanced photosynthetic efficiency through improved chlorophyll synthesis and optimized chlorophyll a/b ratios, promoted nitrogen storage and internal cycling with significantly higher fertilizer-derived nitrogen allocation to structural organs, and stimulated the development of a more efficient root architecture with substantial increases in root volume. These integrated physiological improvements collectively enhanced biomass production and nitrogen accumulation, while simultaneously reducing environmental risks and input costs. The superior performance of MN treatment underscores that balanced nitrogen nutrition, rather than maximum nitrogen input, is crucial for sustainable rubber tree cultivation.

Overall, from a practical perspective, MN application offers compelling economic and environmental advantages: it reduces fertilizer inputs by 30-40% compared to conventional high-nitrogen regimes while maintaining optimal growth, directly lowering production costs and minimizing nitrogen leaching and greenhouse gas emissions. The improved root architecture and nitrogen storage dynamics further enhance plantation resilience to environmental stresses and support more consistent latex production. Future research should focus on validating these findings in field conditions across multiple seasons and different rubber tree clones, with particular emphasis on long-term effects on latex yield and ecosystem impacts. These findings provide a scientific foundation for transitioning toward more sustainable, climate-smart rubber cultivation systems that balance productivity with environmental stewardship.

## Data Availability

The datasets presented in this article are not readily available because The original contributions presented in the study are included in the article/supplementary material, further inquiries can be directed to the corresponding author. Requests to access the datasets should be directed to YZ, zhyfa02@163.com.
